# A 29-year retrospective analysis of koala rescues in New South Wales, Australia

**DOI:** 10.1371/journal.pone.0239182

**Published:** 2020-10-28

**Authors:** Renae Charalambous, Edward Narayan

**Affiliations:** 1 School of Science, Western Sydney University, Penrith, New South Wales, Australia; 2 School of Agriculture and Food Sciences, Faculty of Science, The University of Queensland, St Lucia, Queensland, Australia; 3 Queensland Alliance for Agriculture and Food Innovation, The University of Queensland, St Lucia, Queensland, Australia; Sichuan University, CHINA

## Abstract

The koala (*Phascolarctos cinereus*) is currently listed by both the IUCN and the Australian Governments’ Threatened Species Scientific Committee as vulnerable to extinction with an overall decreasing population trend. It is unknown exactly how many koalas remain in the wild, but it is known that habitat fragmentation and bushfires have ultimately contributed to the decline of the koala all over Australia. This novel study is a retrospective analysis of data over a 29-year period (1989–2018) using records for 12,543 sightings and clinical care admissions for wild koalas from the major koala hot-spots (Port Stephens, port Macquarie and Lismore) in New South Wales, Australia. This study aims to understand the long-term patterns and trends of key stressors that are contributing to the decline of koalas in New South Wales, and the synergic interactions of factors such as rescue location, sex and age of the koala, and if their decline is influenced progressively by year. The main findings of this retrospective analysis indicated that between all 3 rescue sites, the most common prognosis was disease, the most common disease was signs of chlamydia, and the most common outcome was release. The location where the highest number of koalas were found prior to being reported as sighted or admitted into clinical care was within the regional area of Lismore. Furthermore, sex was not a discriminating factor when it came to prognosis or outcome, but age was significant. Finally, incidents of disease were found to increase over long-term, whereas release decreased over time and euthanasia increased. The wealth of data available to us and the retrospective analysis enabled us in a way to ‘zoom out’ and reveal how the key environmental stressors have fluctuated spatially and temporally. In conclusion, our data provides strong evidence of added pressures of increased human population growth in metropolitan zones, which increases risks of acute environmental trauma and proximate stressors such as vehicle collisions and dog-attacks as well as increased sightings of virtually healthy koalas found in exposed environments. Thus our ‘zoom out’ approach provides support that there is an urgent need to strengthen on-ground management, bushfire control regimes, environmental planning and governmental policy actions that should hopefully reduce the proximate environmental stressors in a step wise approach. This will ensure that in the next decade (beyond 2020), NSW koalas will hopefully start to show reversed trends and patterns in exposure to environmental trauma and disease, and population numbers will return towards recovery and stability.

## Introduction

In theory, Australia should have relatively few conservation concerns; its national population density is low (~3km^2^) by global standards (~50km^2^), most of the continent remains sparsely settled and little modified, and the nation is relatively affluent [1]. However, since European Settlement in 1788, 30 mammal species endemic to Australia have become extinct, with 55 others experiencing a worsened conservation status [2]. This statistic is primarily attributed to the fact that humans alter areas of pristine habitat that is rich with biodiversity, so as to accommodate rapid population growth [2].

One of Australia’s most iconic animal species is the koala (*Phascolarctos cinereus*), and can be found in Australian sclerophyll forests and woodlands in Queensland, New South Wales, Australian Capital Territory, Victoria and South Australia [3]. Koalas have a widespread distribution, however this national icon have become subject to considerable population decline throughout all of Australia [3, 4]. The Australian Koala Foundation (AKF) estimate that there could be less than 80,000 wild koalas remaining in Australia, whereas a report by the Chief Scientist of Australia estimate this figure to be around 330,000. Despite inconsistencies relating to population parameters, koalas are listed as “vulnerable to extinction” by both the IUCN (International Union for the Conservation of Nature) in 2014, and the Australian Governments’ Threatened Species Scientific Committee in 2012. Habitat fragmentation is continually described as the reason koalas are experiencing population decline [3, 4], however according to the IUCN, bushfires were listed as the predominant threat associated with this species [5]. Moreover, the removal of suitable tree species in an animals preferred ecosystem is known as habitat fragmentation, and this often results in the susceptibility of that animal to disease through prolonged exposure to stressors [3, 4, 6]. Between 1997 and 2013 in Queensland, Australia, 66.3% of koalas admitted into clinical care were diagnosed with chlamydia disease or presented with signs consistent with chlamydia disease [7]. Infectious pathogens that rely on frequency-dependent transmission, such as chlamydia disease, can influence population dynamics by increasing mortality from wasting and blindness, and decreasing population recruitment through impairment of reproduction [7]. Moreover, in 1998–1999, approximately 1,100 koalas were captured and translocated to the Framlingham Forest located in Victoria, Australia in a bid to intervene with overgrazing in other areas of the state [8]. In January 2007, a deliberately lit bushfire tore through the Framlingham Forest, and it is unknown exactly how many koalas were affected by this fire [8]. Volunteers rescued 147 injured koalas but the fates of only 87 animals were recorded; 38 koalas were taken in by wildlife carers, 33 were subsequently released, 2 went to a sanctuary and 14 were euthanised [8]. In late 2019, Australia experienced unprecedented bushfires, and there is no evidence yet to suggest how many koalas were affected by this fire, however it is estimated that the effect to New South Wales koala populations was catastrophic. It is demonstrated that both habitat fragmentation and bushfires have ultimately contributed to the decline of the koala species all over Australia [3–8].

This study is a retrospective analysis whereby records for 12,543 sightings and clinical care admissions for koalas in New South Wales, Australia, were studied over a 29-year period (1989–2018). Previously, data of this comprehensive nature from New South Wales koala populations was unavailable in the published scientific domain. Data in the way of records from main koala hot-spots throughout New South Wales were collected by collaborating with 3 major wildlife rescue groups including Port Stephens Koalas in Port Stephens, Port Macquarie Koala Hospital in Port Macquarie and Friends of the Koala in Lismore. Our analysis is in a way a ‘zoom out’ approach whereby we hope to show the longitudinal patterns and trends of (across years and decades) key stressors that are contributing to the decline of koalas in New South Wales, and the synergic interactions of factors such as location, sex, age, and if their decline is influenced progressively by year. It is hypothesised that there will be no difference between the factors (location found [Port Stephens, Port Macquarie and Lismore], sex [male or female], and age [adult, joey, juvenile and mature]), but that there will be a significant difference in year admitted into care [1989–2018], as bushfires and habitat fragmentation are major threats that have increased in severity over time with an increase in human population.

## Methods

Research was performed in accordance with relevant guidelines and regulations. Formal approval was granted by the Western Sydney University Animal Care and Ethics (ACEC) Committee (approval/protocol number: A12373).

## Data analysis

Records from both sightings and clinical care admissions for 12,543 koalas were collected from either Port Stephens Koalas in Port Stephens, Port Macquarie Koala Hospital in Port Macquarie or Friends of the Koala in Lismore. Records that were able to be collected ranged from 1989 to 2018.

Port Stephens Koalas is located at 562 Gan Gan Road, One Mile (GPS Coordinates: -32.763792, 152.115904). At maximum capacity, the hospital can hold 20 koalas at one time, however when koala numbers exceed capacity, long-term carers are able to care for koalas in a foster home setting until they are able to be released. Port Macquarie Koala Hospital is located within the Roto House Historical Site on Lord Street, Port Macquarie (GPS Coordinates: -31.442102, 152.919167). At maximum capacity, the hospital can hold 100 koalas at one time. Friends of the Koala is located at 23 Rifle Range Road, East Lismore (GPS Coordinates: -28.820714, 153.302499). At maximum capacity, the hospital can hold 25 koalas at one time, however when koala numbers exceed capacity, long-term carers are able to care for koalas in a foster home setting until they are able to be released.

The information recorded for each koala includes: **sex** (male, female, unknown [some records were sightings only so sex could not be determined]), **age** (joey, juvenile, adult, mature and unknown [some records were sightings only so age could not be determined]), **location** (this refers to what suburb in New South Wales that the koala was either observed at, or rescued from), **prognosis** (this refers to the reason for the koala being recorded as a sighting or being admitted into clinical care and include appearing healthy on assessment, attacked by cattle, collared for tracking, diagnosed with disease, dead on arrival, attacked by a dog, caught in a fire, harassed by humans, hit by a car, orphan, attacked by a snake, unknown, or displaced in unsuitable environment), **outcome** (this refers to the conclusion for that koalas stay in clinical care and include released back to the wild, euthanised, dead on arrival, died in care, trapped and relocated, unknown, transfer to a different captive facility, still in care, escaped from care/self-release [note: some outcomes included providing advice to the member of public, recording a sighting of a koala, unable to capture, and member of the public would not hand the koala over to the carer; although these koalas were not admitted into care, it is still relevant for understanding population dynamics]), and **year** (this refers to what year the koala was either sighted or entered clinical care).

The information recorded from both sightings and clinical care admissions (sex, age, location, prognosis, outcome and year) were entered into a Microsoft^®^ Excel spreadsheet. The spreadsheet was then analysed in IBM SPSS Statistics^®^. Two tests were run for each variable (8 tests in total) in the hypothesis so as to determine trends in the data (independent variables against dependent variables). Moreover, these tests included a descriptive statistical analysis and a generalised linear model. Sex, age, location and year (independent variables) were individually plotted against both the admission prognosis and outcome (dependant variables). Results (in numerical value) were transformed into proportions to enable effective comparisons between the variables. Additionally, the dependant variable [prognosis] was graphed against one of the results of the independent variable [disease] to measure the frequency of specific diseases in New South Wales koala populations. Again, the results (in numerical value) were transformed into proportions to enable effective comparisons between the variables.

ArcGIS, a geographic information system, was used to map the distribution trends of all koalas from the records of both sightings and clinical care admissions. This was performed by extracting location information on the aforementioned Microsoft^®^ Excel spreadsheet to determine the postcode associated with each koala record. The number of koalas admitted within each postcode was then transcribed into comma-separated documents (CSV) on Microsoft^®^ Excel. The CSV sheets were then uploaded separately as a base layer on ArcGIS, and a dot distribution map was generated and koala distribution trends were attained.

It is important to note that due to the stochastic nature of admissions into clinical care based on altered population density, the number of koalas admitted into each wildlife rescue group, and different management strategies by each wildlife rescue group, the available temporal data was not uniform at each location. As a result of this, proportional data was used when comparing locations while most of the analyses focused on trends within individual sites.

## Results

Once the information recorded from both sightings and clinical care admissions were entered into a Microsoft^®^ Excel spreadsheet and results were generated to compare the independent variables with the dependent variables, the most significant findings were extracted for this study.

## Distribution trends

### Port Stephens Koalas in Port Stephens

The suburbs where the highest number of koalas were found prior to being reported as sighted or admitted into clinical care were Salamander Bay (335 koalas), Anna Bay (276 koalas) and One Mile (195 koalas).

### Port Macquarie Koala Hospital in Port Macquarie

The suburbs where the highest number of koalas were found prior to being reported as sighted or admitted into clinical care were Port Macquarie (430 koalas), Armidale (14 koalas) and Limeburners Creek (13 koalas).

### Friends of the Koala in Lismore

The suburbs where the highest number of koalas were found prior to being reported as sighted or admitted into clinical care were Goonellabah (1,219 koalas), Lismore (1,004 koalas) and Wyrallah (444 koalas).

## Trends in prognosis

**Fig 1:**
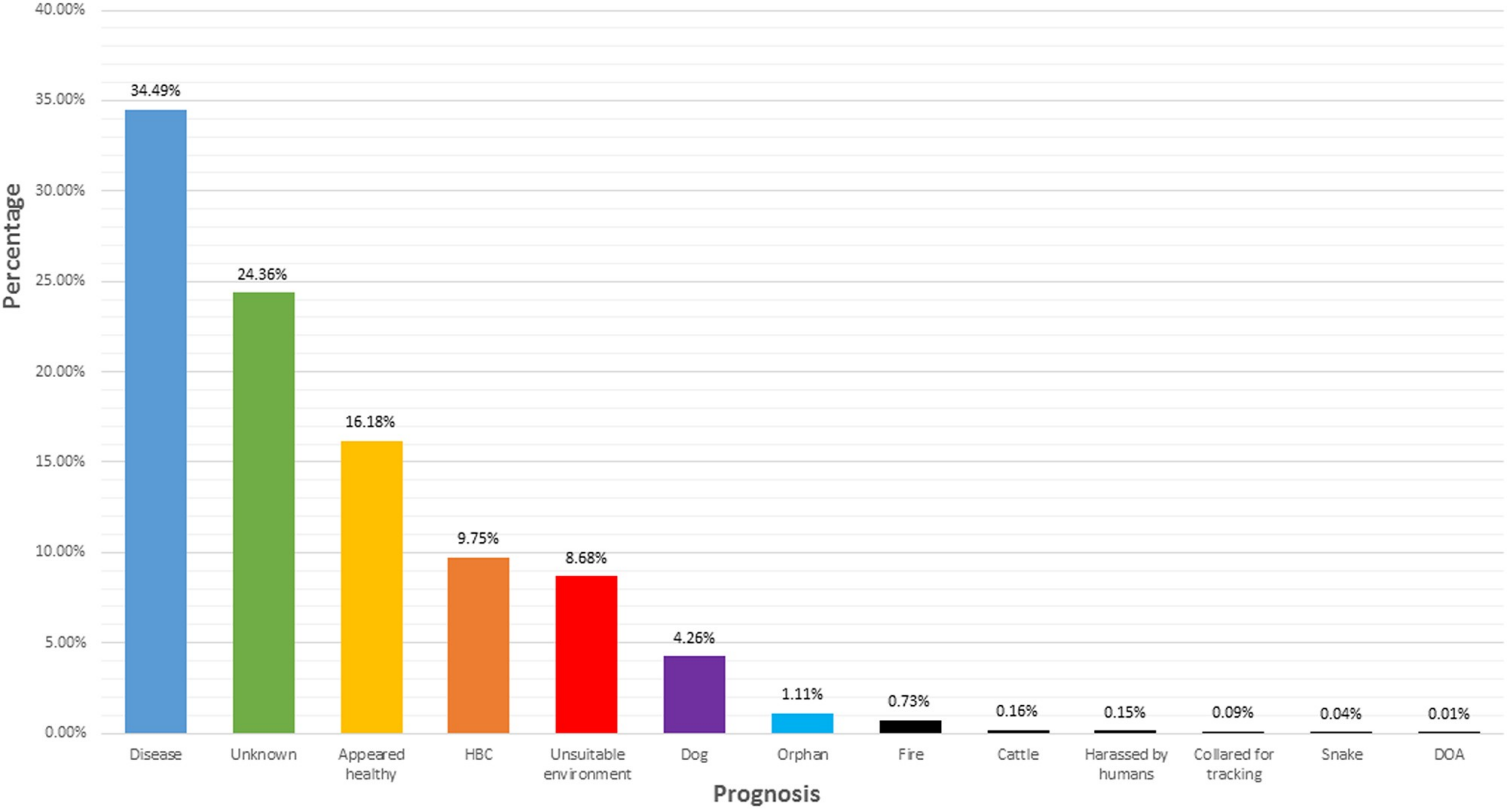
Prognosis recorded for each koala (as per sighting or admission into clinical care) for 12,543 koalas, logged as a percentage.

Prognosis refers to the reason that each koala was recorded as a sighting or the reason that the koala was admitted into clinical care. Of all 12,543 records over the 3 wildlife groups studied in New South Wales, **34.5%** of koalas were recorded as having a *disease*, **24.4%** were recorded as having an *unknown prognosis*, and **16.2%** were recorded as *appeared healthy* (Fig 1).

## Trends in disease as a prognosis

**Fig 2:**
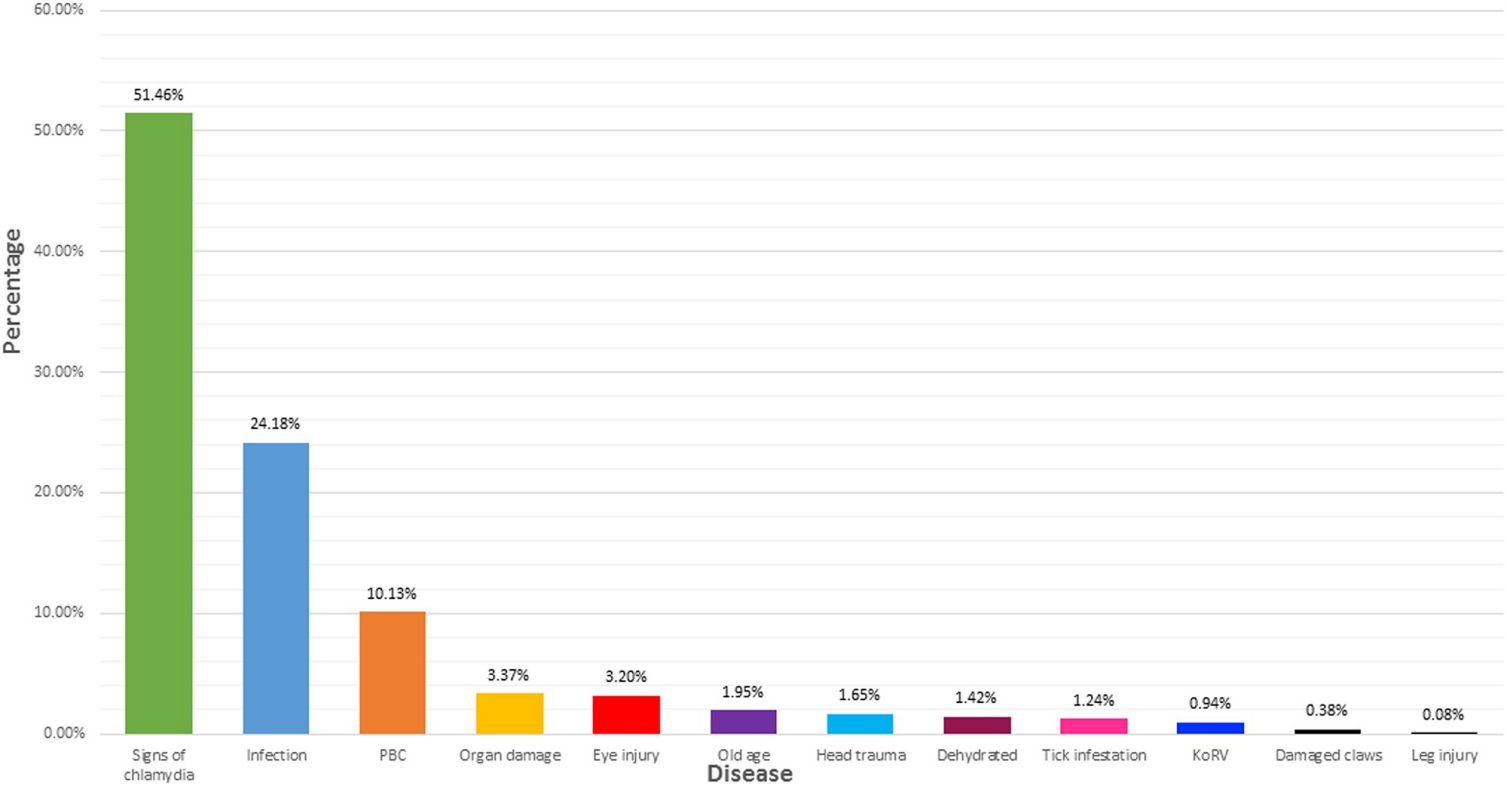
Disease recorded for each koala (as per sighting or admission into clinical care) for koalas with the prognosis disease, logged as a percentage.

The highest recorded prognosis for koalas over all 3 wildlife rescue groups was *disease* **(34.5%).** The diseases (or symptoms thereof) that koalas were diagnosed from includes: **having signs of chlamydia** (signs include having a wet bottom and/or conjunctivitis and is not limited to an actual diagnosis of chlamydia), **infection** (includes confirmed infection to 1 or many areas of the body), **poor body condition** (refers to too little energy being consumed to meet the animals energy requirements, leading to being underweight), **organ damage** (includes varying levels of damage to 1 or many organs), **eye injury** (injuries damage to 1 or both eyes and can involve impaired vision or blindness), **old age** (refers to the mature age category), **head trauma** (experiencing varying levels of trauma to the head), **dehydration**, **tick infestation**, **koala retrovirus**, **damaged claws** (refers to varying levels of injury to 1 or many claws), and **leg injury** (refers to varying levels of injury to 1 or both legs causing limited to no ability to walk). Of the koalas who were recorded as having a disease as their prognosis, **51.5%** had *signs of chlamydia*, **24.2%** had an *infection*, and **10.1%** had *poor body condition* (Fig 2).

## Trends in outcome

**Fig 3:**
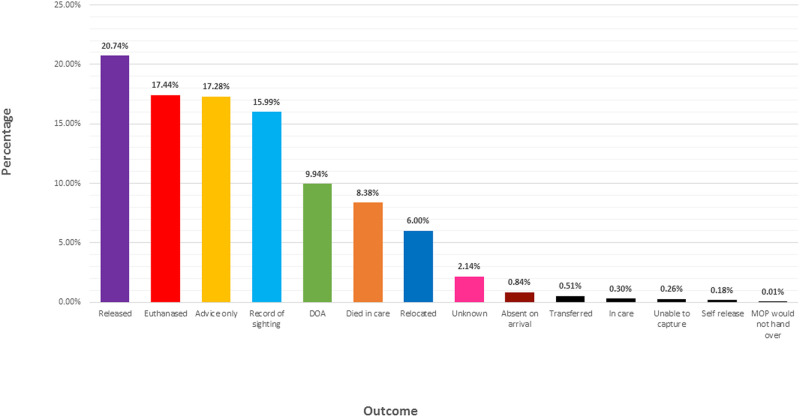
Outcome recorded for each koala (as per sighting or admission into clinical care) for 12,543 koalas, logged as a percentage.

Outcome refers to the conclusion for that koalas stay in clinical care or why they were not admitted into clinical care. Of all 12,543 records over the 3 wildlife groups studied in New South Wales, **20.7%** of koalas were *released*, **17.4%** were *euthanised*, and **17.3%** required *advice only* (Fig 3).

## Comparing prognosis and outcome with age, sex, location and year

### Age

Age categories for koalas were grouped according to life stages. A **joey** is a koala between the age of birth to 6 months old, a **juvenile** is between 6 months and 1 year, an **adult** describes sexual maturity and is between 1 year and 7 years, and a **mature** koala is over 7 years old [note: see discussion for an in-depth review of life stages]. The most common prognosis among all records collected was *disease*, and the age that was most affected by this prognosis was *mature* koalas (42.2%). The most common outcome among all records collected was released, and the age that was affected most by this outcome was *mature* koalas (45.8%).

### Sex

Sex categories for koalas were grouped by male, female or unknown. The unknown category is due to the fact that some records were sightings only so sex could not always be determined. The most common prognosis among all records collected was *disease*, and there was no outstanding sex that was most affected by this prognosis (*female* koalas [38.9%] and *male* koalas [38.3%]). The most common outcome among all records collected was released, and again, there was no outstanding sex that was most affected by this outcome (*female* koalas [33%] and *male* koalas [30.4%]).

### Location

Location categories for koalas were grouped according to the wildlife rescue group they were admitted to, and they include Port Stephens, Port Macquarie or Lismore. The most common prognosis among all records collected was *disease*, and the location that was most affected by this prognosis was *Lismore* (37.1%). The most common outcome among all records collected was released, and the location that was affected most by this outcome was *Port Macquarie* (71.4%).

### Year

**Fig 4:**
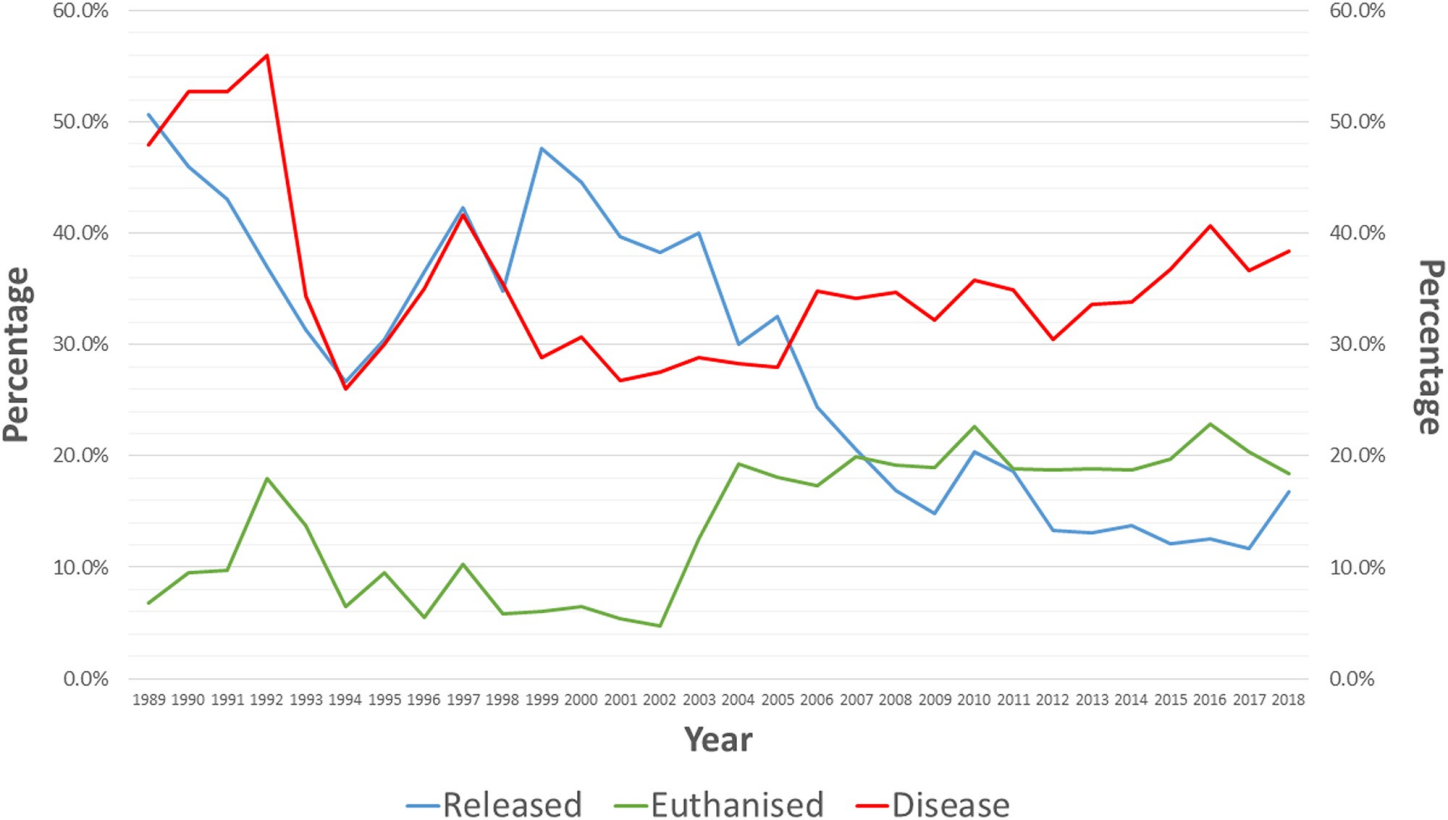
Data recorded for each koala (as per sighting or admission into clinical care) for koalas with the prognosis disease, against koalas with the outcome released, and the outcome euthanised logged as a percentage over time (1989–2018).

Records on koalas were collected from each wildlife rescue group from the year starting in 1989 to the year ending in 2018. The most common prognosis among all records collected was *disease*, and the year that this prognosis was highest was *1992* (56%) (Fig 4). Trends in disease were continually fluctuating over the 29-year period, and after a significant decrease in disease from 1992 to 1994, the prognosis has been fairly consistent since 2000 (Fig 4). The most common outcome among all records collected was released, and the year that was affected most by this outcome was *1989* (50.7%) (Fig 4). Trends in release peaked in 1989, 1997 and 1999, but has been decreasing since 2000 (Fig 4).

## Discussion

The aim of this study is to understand the patterns and trends of key stressors that are contributing to the decline of koalas in New South Wales, and the synergic interactions of factors such as location, sex, age, and if their decline is influenced progressively by year. It is hypothesised that there will be no difference between the factors (location found [Port Stephens, Port Macquarie and Lismore], sex [male or female], and age [adult, joey, juvenile and mature]), but that there will be a significant difference in year admitted into care [1989–2018], as bushfires and habitat fragmentation are major threats that have increased in severity over time with an increase in human population.

Habitat is a species-specific concept defined as “the resources and conditions present in an area that produce occupancy” [9]. Despite there being approximately 900 species of eucalypt in Australia, koalas will feed on a select few and depending on their locality, will limit their diet to just 10 species. Studies have shown that there is a higher-frequency of visitation by koalas in New South Wales to eucalypt species such as the manna gum (*Eucalyptus viminalis*) [10]. The manna gum is found in New South Wales, south-eastern Queensland, Victoria, South Australia and Tasmania, and is frequently harvested for its wood which is widely used in Australia. Although rates of deforestation have reduced over time, habitat clearance continues to exert pressure on Australian biodiversity, and koalas are no exception [11]. Recent statistics show that between 2010 to 2014, 297,482.125 hectares of land rich in native eucalypt species such as the manna gum was cleared in New South Wales alone [11]. The leading cause for habitat clearance is influenced by human use, and primarily includes grazing of natural vegetation (animal agriculture and crop growth) [12]. The suburbs where most koalas were found prior to being reported as sighted or admitted into clinical care were Goonellabah (1219 koalas), Lismore (1004 koalas) and Wyrallah (444 koalas), and those suburbs are all located in the regional area of Lismore. According to the Australian Bureau of Statistics, agricultural production in regional areas such as Lismore is a key contributor to the economy, with the total value of agricultural production growing exponentially. For example, in 2001 agricultural production in Lismore was worth $34,236 million, and by 2006, it had risen to $38,527 million. It is therefore not surprising that the suburbs where most koalas were found prior to being reported as sighted or admitted into clinical care were within the regional city of Lismore, where agricultural production is high and therefore so is deforestation of ideal koala habitat such as the eucalypt species, manna gum.

Literature suggests koala populations are declining due to habitat clearance influenced by human use, and primarily includes grazing of natural vegetation (animal agriculture and crop growth) [12]. Any disturbance to an animals habitat activates the physiological stress response [13], and if said stressors do not cease, the excessive production of glucocorticoids can leave the animal with a compromised immune system and therefore likely to contract a disease [4]. Of all 12,543 records over the 3 wildlife groups studied in New South Wales, 34.5% of koalas were recorded as having a disease as their prognosis (Fig 1). Of the koalas who were recorded as having a disease, 51.5% had signs of chlamydia (Fig 2). *Chlamydia pecorum* is an infectious bacterial pathogen that operates as a significant threat to koala conservation [14, 15]. Chlamydia is primarily a sexually transmitted infection in koalas, however there is anecdotal evidence for vertical transmission (transmission of a pathogen from an infected mother to baby either during birth, or after birth) [14]. Efforts to understand this disease have found ocular infections of chlamydia can lead to debilitating blindness, while urogenital tract infections of chlamydia can lead to cystitis and/or ascending infections of the reproductive tract and sterility [16]. In the wild, chlamydia in koalas can be identified through red, inflamed eyes and a brown, wet bottom [17]. Koalas infected with this disease often starve to death as the ocular infection creates proliferative inflamed conjunctival tissue that can grow over the cornea of the eye, rendering the koala blind [18]. Depending on the stage of chlamydia, koalas admitted into clinical care can be administered with antibiotics as a treatment for the disease, although keeping in mind that this can adversely affect the gut microflora and health of the animal [17]. Successful treatment of a koala with chlamydia means that animal can be released back into the wild after a full recovery, and 20.7% of the koalas in this study were successful released back into the wild after treatment in clinical care (Fig 3). A late diagnosis of chlamydia may require euthanasia on the grounds of welfare, as the disease is incredibly painful for the infected koala and often leaves them with long-term health implications [17]. It is therefore not surprising that 17.4% of the koalas in this study were euthanised (Fig 3).

Variation in age demonstrates specific selection pressures that can have a fundamental influence on animal survival [19]. This is due to the fact that fitness requirements differ between age classes in distinctive ways [19]. When koalas are born, they are roughly 2cm long and remain blind and hairless in their mothers’ pouch, feeding off milk from a teat. At this stage, and until they are 6 months old, they are referred to as a joey [20]. From the age of 6 months to 1 year, the joey has transitioned into a juvenile and begins to spend time out of the mothers pouch by cuddling onto her stomach for warmth and shelter, or ride on her back [20]. During this time, the mother will produce a substance called “pap”, which is a micro-organism necessary for making digestion for her juvenile koala possible [20]. After 1 year, the juvenile has transitioned into an adult and is expected to become independent and begins mating [20]. Sexual maturity in female koalas is at 15 months, whereas sexual maturity in male koalas is at 24 months [20]. Once 7 years and above, the adult koala has transitioned into a mature koala and are well beyond sexual maturity [20]. Once a koala is an adult and becomes independent from its mother, survival becomes harder as they have to protect themselves from predators such as cars, dogs, and humans [20]. Adult koalas are frequently moving with the aim of finding a food source or someone to mate with, and the potential to cross roads with cars and enter landscapes with dogs and other predators is high at this time [4]. A study in koalas within south-eastern Queensland found the predominance of mature aged koalas when measuring disease in koala populations [21]. The results of the south-eastern Queensland koalas coincide with the results of this study where the mature age group was most affected by the prognosis disease (42.2%). It is likely that the reason that mature koalas are most affected by disease compared to any other age group is due to increased mobility during breeding season [22, 23]. Adult koalas who are sexually mature will fight for dominance within a group of female koalas, and the mature koalas are forced to move along and find refuge elsewhere, therefore increasing their chance of crossing roads with cars and entering landscapes with dogs and other predators [4, 22, 23]. The most common outcome among all records collected was released, and the age group that was affected most by this outcome was mature koalas (45.8%). It is likely that despite the fact that mature koalas are forced out by adult koalas and left to find refuge elsewhere, they make up a large proportion of koalas being admitted into care and treated, therefore allowing them to be released back into suitable habitat.

Additional to age, variation in sex can demonstrate specific selection pressures that can have a fundamental influence on animal survival [19]. The results of this study however found this not to be the case, as the most common prognosis among all records collected was disease, and there was no outstanding sex that was most affected by this prognosis (female koalas [38.9%] and male koalas [38.3%]). Furthermore, the most common outcome among all records collected was released, and again, there was no outstanding sex that was most affected by this outcome (female koalas [33%] and male koalas [30.4%]). A study in koalas within south-eastern Queensland found that depending on age, sex often affected prognosis and outcome in koalas, however this was not consistently the case [21]. For example, female koalas overall were more likely to be affected by co-morbidities (more than 1 disease or health problem) compared to male koalas, but when age was considered, female and male mature aged koalas were equally as likely to be affected [21]. It is likely that sex was not prejudiced to rates of disease or release in this study, as species vulnerability and susceptibility to stress does not discriminate, and the extent at which survival is threatened for koalas through habitat destruction is critical [24].

The most common prognosis among all records collected was disease, and the location that was most affected by this prognosis was Lismore (37.1%). As previously mentioned, the suburbs where most koalas were found prior to being reported as sighted or admitted into clinical care were within the regional city of Lismore. This is due to agricultural production being high within that area, causing rapid deforestation of ideal koala habitat such as the eucalypt species, manna gum. Loss of habitat is known to activate the physiological stress response [13], and this produces an excess of glucocorticoids that can leave the animal with a compromised immune system and therefore likely to contract a disease [4]. The most common outcome among all records collected was released, and the location that was affected most by this outcome was Port Macquarie (71.4%). At maximum capacity, the Port Macquarie Koala Hospital can hold 100 koalas at one time, compared with 20 at Port Stephens Koalas and 25 at Friends of the Koala. It can be assumed that the outcome release was highest at the location in Port Macquarie due to their increased capacity to hold more koalas under veterinary care at any given time.

Records on koalas were collected from each wildlife rescue group from the year starting in 1989 to the year ending in 2018. The most common prognosis among all records collected was disease, and although this prognosis continually fluctuated over the 29-year period, it gradually increased over time (Fig 4). The most common outcome among all records collected was released, and trends in this outcome display a peak in 1989, 1997 and 1999, but decrease from 2000 (Fig 4). During the 1990s, the Australian population was estimated at 17,041,431 people, and by 2018 was estimated at 24,772,247 people. The difference of 7,730,816 people over a 28-year period is dramatic, especially since it is argued that the environment has officially reached the upper limits of human population growth [25]. With human population growth comes the need to create more space to build homes and grow food, and thus the necessity to alter areas of pristine koala habitat [2]. Furthermore, when bushfires occur, they are known to wreak havoc on biodiversity [26]. If not victim to the fire itself, bushfires often leave animals that are otherwise full of vitality threatened by post-fire famine, and the risk of starvation is high [26]. This threatening survival process is compared to the guillotine, an apt metaphor for the survival process for biodiversity post bushfire [26]. Habitat fragmentation and bushfires often results in the susceptibility of animals to disease through prolonged exposure to stressors [3, 4, 6, 26], and with the human population increasing exponentially, so too does the risk of bushfires and potential disease in koalas, leading to their inability to recover and be released.

As previously mentioned, bushfires and habitat fragmentation are major threats to koalas that have increased in severity over time with an increase in human population. Despite this, records for sightings and clinical care admissions in this study listed 0.7% of koalas being admitted into care for the prognosis fire, and 8.7% for the prognosis unsuitable habitat (Fig 1). It is likely that an understanding of the physiological stress response will explain these results, including why the leading prognosis was instead disease (34.5%) (Fig 1). Essentially, stress is broadly defined as a change in psychological, physiological and/or physical well-being of a living organism as a result of exposure to any biological and/or environmental factor that acts as a stressor/challenge to regular capacity [27]. Once the hypothalamic-pituitary-adrenal (HPA) axis is activated as a response to stress, a complex negative-feedback system begins as a way to maintain allostasis [28, 29]. This system is not inherently detrimental, however allostatic overload and the production of glucocorticoids by the HPA axis can lead to changes in immunological processes that influence the onset of disease [27]. The stress associated with bushfires and habitat fragmentation, which often induce stress gradually over time, have likely resulted in the leading prognosis being disease. As previously mentioned, 51.5% of koalas with the prognosis disease had signs of chlamydia (Fig 2). These results are not surprising as the most clinically significant cause of infectious disease in koalas is the bacterial parasite chlamydia [30]. The symptoms associated with chlamydia include ocular disease (discharge, conjunctival and corneal inflammation) as well as urogenital tract disease (cystitis, urinary incontinence and fibrosis causing infertility) [30]. Depending on the stage, koalas admitted into clinical care can be administered with antibiotics as a treatment for chlamydia, although as mentioned earlier, this can adversely affect the gut microflora and health of the animal [17]. Successful recovery can lead to release of that koala back into the wild, however the number of koalas successfully released has decreased since the early 2000’s, whereas the number of koalas euthanised has increased (Fig 4). This would suggest that koalas admitted into clinical care are suffering with late stage chlamydia and that koala could be infertile or incurable.

Finally, some study limitations should be noted. The number of koalas admitted into each wildlife rescue group, and different management strategies by each wildlife rescue group meant that data was not uniform at each location. When results were analysed, proportional data was used to compare locations and trends. Furthermore, the majority of records were collected from each location, however due to time constraints, some records were excluded and so total records for sightings and clinical care admissions may be higher. Additionally, some records are listed as “unknown” due to the gaps in the rescue groups’ data and not having that information on record. Unfortunately, this information could have fallen into other categories such as “disease” or “released” however that remains unknown.

## Conclusion

This study is a retrospective analysis whereby records for 12,543 sightings and clinical care admissions for koalas in New South Wales, Australia, were studied over a 29-year period (1989–2018). Previously, data of this comprehensive nature from New South Wales koala populations was unavailable in the published scientific domain. Data in the way of records from main koala hot-spots throughout New South Wales were collected by collaborating with 3 major wildlife rescue groups including Port Stephens Koalas in Port Stephens, Port Macquarie Koala Hospital in Port Macquarie and Friends of the Koala in Lismore. Furthermore, this study aims to understand the patterns and trends of key stressors that are contributing to the decline of koalas in New South Wales, and the synergic interactions of factors such as location, sex, age, and if their decline is influenced progressively by year.

It was hypothesised that there would be no difference between prognosis and outcome with the factor of location found. This hypothesis was disproved as the location where the highest number of koalas were found prior to being reported as sighted or admitted into clinical care was within the regional area of Lismore. These results were due to the fact that regional areas such as Lismore depend heavily on agricultural production. Demands have increased with human population growth, and areas that koalas occupy are being repurposed for agricultural production.

It was hypothesised that there would be no difference between prognosis and outcome with the factor of sex. This hypothesis was proved as sex did not discriminate between the prognosis disease (female koalas [38.9%] and male koalas [38.3%]) or the outcome release (female koalas [33%] and male koalas [30.4%]). It is likely that sex was not prejudiced to rates of disease or release in this study, as species vulnerability and susceptibility to stress does not discriminate, and the extent at which survival is threatened for koalas through habitat destruction is critical.

It was hypothesised that there would be no difference between prognosis and outcome with the factor of age. This hypothesis was disproved as the mature age group was most affected by the prognosis disease (42.2%), and the outcome released (45.8%). This is due to the fact that adult koalas who are sexually mature will fight for dominance within a group of female koalas, and the mature koalas are forced to move along and find refuge elsewhere. This increases their chance of crossing roads with cars and entering landscapes with dogs and other predators. By the same account, mature aged koalas are entering care and are able to be treated, therefore allowing them to be released back into suitable habitat.

It was hypothesis that there would be a significant difference between prognosis and outcome with the year admitted into care. This hypothesis was proved as the prognosis disease and the year admitted into care fluctuated significantly, and ultimately increased as each year progressed. Furthermore, the outcome released and the year admitted into care ended up decreasing significantly as each year progressed, despite peaking in 1989, 1997 and 1999. This is due to the fact that an increase in both human population growth and the prevalence of bushfires have placed a significant hinderance on koalas’ ability to survive.

## Supporting information

S1 Data Raw data for records detailing 12,543 sightings and clinical care admissions for koalas in New South Wales, Australia, over a 29-year period (1989–2018). (XLSX)
