# Correction: A 29-year retrospective analysis of koala rescues in New South Wales, Australia

**DOI:** 10.1371/journal.pone.0292911

**Published:** 2023-10-10

**Authors:** Renae Charalambous, Edward Narayan

The term “prognosis” is used inaccurately throughout the article [1]. The correct term is “reason for sighting or admission” and include appearing healthy on assessment, attacked by cattle, collared for tracking, diagnosed with disease, dead on arrival, attacked by a dog, caught in a fire, harassed by humans, hit by a car, orphan, attacked by a snake, unknown, or displaced in unsuitable environment.

In the Introduction, a reference [2] has been omitted from the second sentence of the last paragraph. The authors stated that the dataset in [1] is different to and is not derived from the dataset in [2]: [1] contains 12,543 records covering the period 1989–2018 from three major koala hospitals throughout New South Wales, whereas [2] contains 3,781 records covering the period 1975–2004 from one major koala hospital within NSW. The authors also stated that the dataset in [1] was collected manually from paperwork held by each koala hospital, as there is no central database for koala hospital admissions in New South Wales.

The *PLOS ONE* Editors and the authors regret that these errors were not identified prior to publication.
